# MoSe_2_-Ni_3_Se_4_ Hybrid Nanoelectrocatalysts and Their Enhanced Electrocatalytic Activity for Hydrogen Evolution Reaction

**DOI:** 10.1186/s11671-020-03368-z

**Published:** 2020-06-16

**Authors:** Pengyuan Wu, Gangyong Sun, Yuanzhi Chen, Wanjie Xu, Hongfei Zheng, Jin Xu, Laisen Wang, Dong-Liang Peng

**Affiliations:** Department of Materials Science and Engineering, Collaborative Innovation Center of Chemistry for Energy Materials, College of Materials, Xiamen University, Xiamen, 361005 China

**Keywords:** Hydrogen evolution reaction, MoSe_2_, Electrocatalysis, Nickel selenides, Hybrid nanostructure

## Abstract

Combining MoSe_2_ with other transition metal dichalcogenides to form a hybrid nanostructure is an effective route to enhance the electrocatalytic activities for hydrogen evolution reaction (HER). In this study, MoSe_2_-Ni_3_Se_4_ hybrid nanoelectrocatalysts with a flower-like morphology are synthesized by a seed-induced solution approach. Instead of independently nucleating to form separate nanocrystals, the Ni_3_Se_4_ component tends to nucleate and grow on the surfaces of ultrathin nanoflakes of MoSe_2_ to form a hybrid nanostructure. MoSe_2_–Ni_3_Se_4_ hybrid nanoelectrocatalysts with different Mo:Ni ratios are prepared and their HER catalytic activities are compared. The results show that the HER activities are affected by the Mo:Ni ratios. In comparison with pure MoSe_2_, the MoSe_2_-Ni_3_Se_4_ hybrid nanoelectrocatalysts having a Mo:Ni molar ratio of 2:1 exhibit enhanced HER properties with an overpotential of 203 mV at 10 mA/cm^2^ and a Tafel slope of 57 mV per decade. Improved conductivity and increased turnover frequencies (TOFs) are also observed for the MoSe_2_-Ni_3_Se_4_ hybrid samples.

## Introduction

Traditional fossil fuels are the main energy sources in our society; however, they are non-renewable and unsustainable, and are causing serious pollution to environment. Among alternative energies, hydrogen energy has been regarded as one of the most promising clean energies because of its ultrahigh energy density [1]. Up to now, the large-scale production of hydrogen is still mainly from fossil fuel sources [2]. Coal gasification and methane steam reforming industrially produce 95% of hydrogen [3]. Hydrogen evolution reaction (HER) has been considered as a promising route to generate high-purity hydrogen [1, 4, 5]. However, the best electrocatalysts for HER in acidic media are still Pt-based and other noble metal materials [6]. Due to their scarcity and high cost, the Pt-based materials are not suitable to be applied in large-scale hydrogen evolution [7].Transition metal dichalcogenides (TMDs), like MoS_2_, MoSe_2_, WS_2_, and WSe_2_, have received intensive attentions owing to their excellent electrochemical properties and earth abundant nature. As a typical layered TMD semiconducting material, MoSe_2_ has a similar structure to graphite, and is formed by Se–Mo–Se layers that are bonded via the van der Waals forces. In addition, MoSe_2_ is more metallic than MoS_2_, and has a lower Gibbs free energy of the hydrogen adsorption onto the edge of MoSe_2_ than MoS_2_, which leads to a higher adsorption of hydrogen [8]. On this account, MoSe_2_ and its hybrids have captured much attention as electrocatalysts for HER.

It is well known that only active sites are effective for HER. For two-dimensional layered nanostructures like TMD nanosheets, the active sites for HER are located along the nanosheet edges [9], whilst the basal surfaces are inert. The conductivity of electrocatalysts is also an important issue for HER. As a kind of semiconductor, the poor electron transport ability of MoSe_2_ compared to noble metals is still limiting its performance in HER [10]. Therefore, the general strategies for improving the activity of TMD catalysts are to enhance the electrical conductivity [11, 12] and increase the active site numbers [12–14]. Meanwhile, designing hybrid structures by integrating different types of semiconductive materials especially TMDs with a preferred orientation is considered to be an important approach to tuning the electronic properties of semiconductive materials [15–17]. Hybrid nanostructures with efficient heterointerfaces can promote rapid interfacial charge transfer, which is pivotal to the electrochemical reactions [18]. Besides, it is well known that three elementary steps, i.e., adsorption, reduction, and desorption, are required to generate hydrogen during the electrochemical reactions [19]. One of the superiority for hybrid materials composing of different chemical components is that they may break through the limitation that many single-component catalysts are not effective for all the three intermediate reaction processes. Recently, some researchers have integrated Ni-based catalysts with MoSe_2_ in various morphologies by using different methods to achieve enhanced HER performances [15, 18, 20]. The combination of MoSe_2_ with Ni selenides to form a hybrid structure may utilize the synergistic effect that arises from the interaction between two heterogeneous components to achieve enhanced electrocatalytic activity. For example, a DFT calculation indicated that the MoS_2(1−*x*)_Se_2*x*_/NiSe_2_ had much lower hydrogen adsorption Gibbs free energy on (100) and (110) planes than pure MoS_2(1−*x*)_Se_2*x*_, which could result in higher coverage of hydrogen at the active sites and therefore achieved outstanding electrocatalytic performances [21].

Herein, we attempt to prepare hybrid nanoelectrocatalysts by growing Ni_3_Se_4_ on the surfaces of flower-like MoSe_2_ seeds which are synthesized via a colloidal method reported in our previous study [22]. Such a seed-induced growing approach offers a facile means to build various TMD hybrid nanostructures. The reason why we select Ni_3_Se_4_ as the hybrid component is that Ni_3_Se_4_ has a higher electrical conductivity than other nickel selenides [23]. In order to investigate the influences of Ni_3_Se_4_ on catalyst’s activity and find out the best composition ratio, we systematically modulated the content of Ni_3_Se_4_ and MoSe_2_, and found that the incorporation of moderate content Ni_3_Se_4_ into the MoSe_2_–Ni_3_Se_4_ hybrid systems can improve the HER performances. Our results suggest that the construction of a hybrid nanostructure of MoSe_2_–Ni_3_Se_4_ is an effective approach to improve the HER performances of pure MoSe_2_.

## Methods/Experimental

### Synthesis of MoSe_2_–Ni_3_Se_4_ Hybrid Nanoelectrocatalysts

The synthesis of MoSe_2_–Ni_3_Se_4_ hybrid nanoelectrocatalysts involved two steps. In the first step, MoSe_2_ seeds were synthesized according to the method reported in our previous study [22]. Briefly, 10 mL of oleic acid (OA, 85%, Aladdin Bio-Chem Technology Co., Ltd.) and 0.4 mmol of molybdenum hexacarbonyl (Mo(CO)_6_, 98%, J&K Scientific Ltd.) were mixed and heated up to 85 °C slowly in argon gas. Subsequently, the temperature of the mixed solution was increased to 200 °C and 6.7 mL of pre-prepared solution containing 1-octadecene (ODE, 90%, Aladdin Bio-Chem Technology Co., Ltd.) and Se (99.999%, J&K Scientific Ltd.) with a Se concentration of 0.15 mmol/mL was injected into the reaction solution using an injecting speed of 0.5 mL/min. When the injection was completed, the reaction was further maintained for 30 min to generate MoSe_2_ seeds. In the next step, the reaction temperature was increased to 300 °C, and a mixture of 3.3 mL solution of ODE and Se, and nickel(II) acetylacetonate (Ni(acac)_2_, 0.2 mmol, 96%, J&K Scientific Ltd.) was injected into the reaction mixtures and kept at 300 °C for 30 min. After cooling down to room temperature, the reaction products were washed with ethanol and hexane, and then undergoing drying at room temperature. The synthesized sample was labeled as Mo2Ni1, denoting that the molar ratio of Mo:Ni in MoSe_2_–Ni_3_Se_4_ hybrid samples is 2:1. Other MoSe_2_–Ni_3_Se_4_ nanohybrid samples with different Mo to Ni ratios were synthesized using the same procedure except that different qualities of mixtures of Ni and Se sources were added in the reaction.

### Characterization

The crystalline phase was characterized using by an X-ray diffractometer (Bruker D8-Advance). Transmission electron microscopy (TEM) images were obtained using a JEM-2100 transmission electron microscope. High-angle annular dark-field (HAADF) imaging and corresponding elemental mapping were performed with a TECNAI F-30 transmission electron microscope. Scanning electron microscopy (SEM) images were acquired using a SU-70 scanning electron microscope. X-ray photoelectron spectroscopy (XPS) data were obtained via a spectrometer (PHI QUANTUM 2000) with Al Kα source.

### Electrochemical Tests

The electrochemical tests were conducted in a standard testing system containing a reference electrode of Ag/AgCl, a graphite rod counter electrode and a glass-carbon working electrode which were connected to an Autolab 302N electrochemical workstation that used H_2_SO_4_ (0.5 M) as electrolyte. To prepare electrocatalyst ink, the synthesized electrocatalysts (4 mg), Ketjenblack carbon black (0.5 mg), and Nafion solution (30 μL) were mixed with ethanol-water solution (1 mL) with an ethanol content of 20 vol%. The mixtures were then ultrasonicated for 30 min. Finally, 5 μL of ink (containing about 20 μg electrocatalysts) was deposited on the glassy carbon electrode to form a film that had a loading of about 0.286 mg/cm^2^ and dried at room temperature. The polarization curves were obtained by using a scan rate of 2 mV s^−1^ at 25 °C from 0.2 to − 0.6 V (versus reversible hydrogen electrode (RHE)). The electrochemical impedance spectroscopy (EIS) data were obtained at frequencies ranging from 0.01 Hz to 100 kHz at – 260 mV. The cyclic voltammetry (CV) test was carried out to obtain the double-layer capacitance (non-Faradaic potential) from 0.1 to 0.2 mV and to calculate the effective surface area of electrode.

## Results and Discussion

**Fig. 1:**
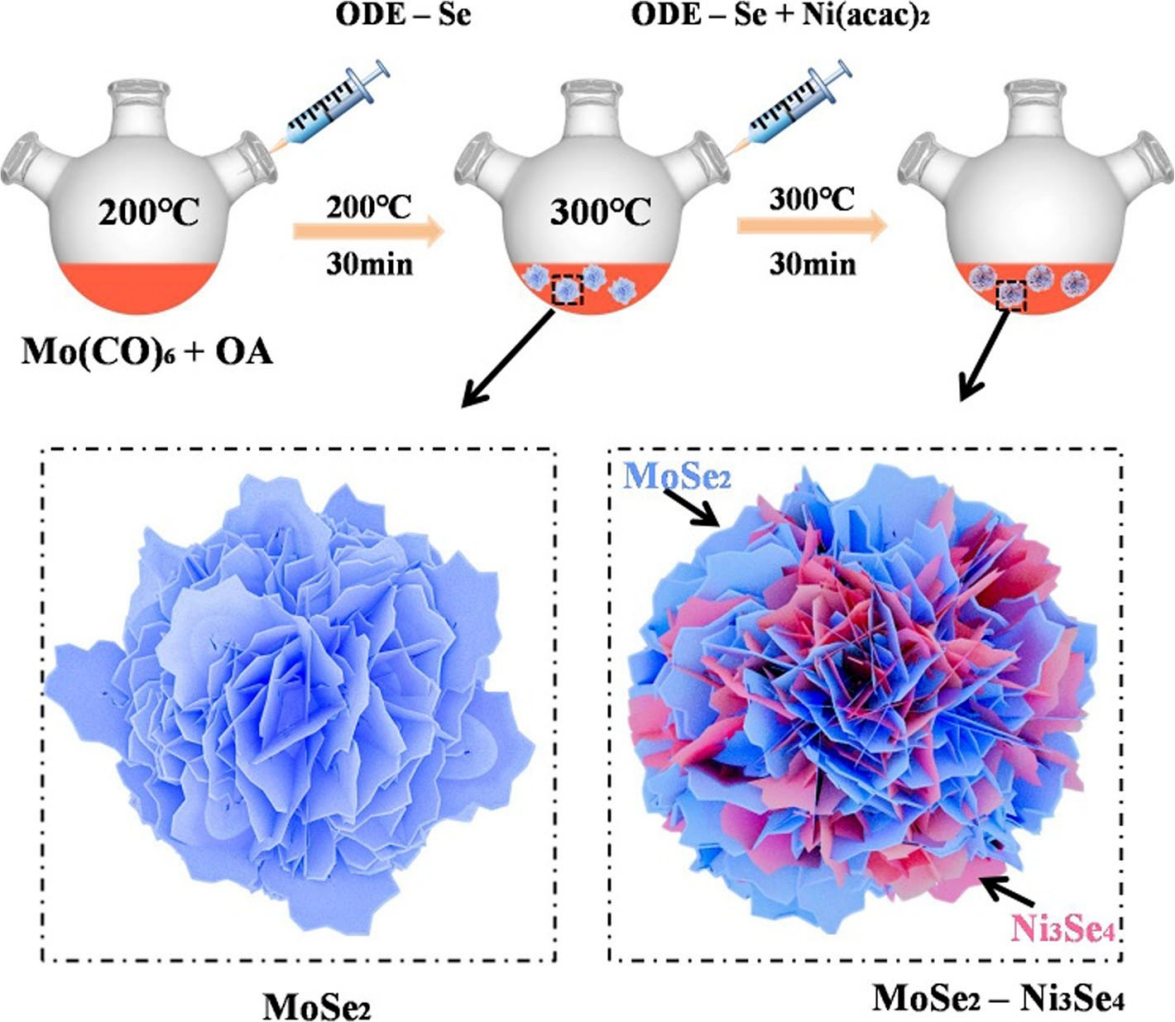
Schematic diagram of the formation of MoSe_2_–Ni_3_Se_4_ hybrid nanoelectrocatalysts

The synthesis of MoSe_2_–Ni_3_Se_4_ hybrid nanoelectrocatalysts is based on a seed-induced strategy in which nanoscale Ni_3_Se_4_ grows in situ on the pre-formed MoSe_2_ seeds (Fig. 1). In the first step, MoSe_2_ seeds were synthesized via the reaction between Mo precursor (Mo(CO)_6_) and Se in the presence of OA in ODE at 200 °C in which process ultrathin MoSe_2_ nanoflakes which were formed during the heating process were further self-assembled into flower-like MoSe_2_ particles [22]. The flower-like morphology with large surface area may facilitate the dispersion and intimate interaction of the second component [24]. After the temperature reached at 300 °C, the solution containing Ni(acac)_2_ and ODE-Se was rapidly injected into the hot reaction mixtures containing MoSe_2_ seeds. At this stage, Ni_3_Se_4_ nucleates and grows on the surface of MoSe_2_ nanoflakes to form MoSe_2_–Ni_3_Se_4_ hybrid nanostructures. This facile synthetic strategy is effective for the synthesis of MoSe_2_–Ni_3_Se_4_ hybrid nanoelectrocatalysts with different Mo:Ni ratios under similar experimental conditions and may be employed to build other MoSe_2_-based hybrid nanoelectrocatalysts.

**Fig. 2:**
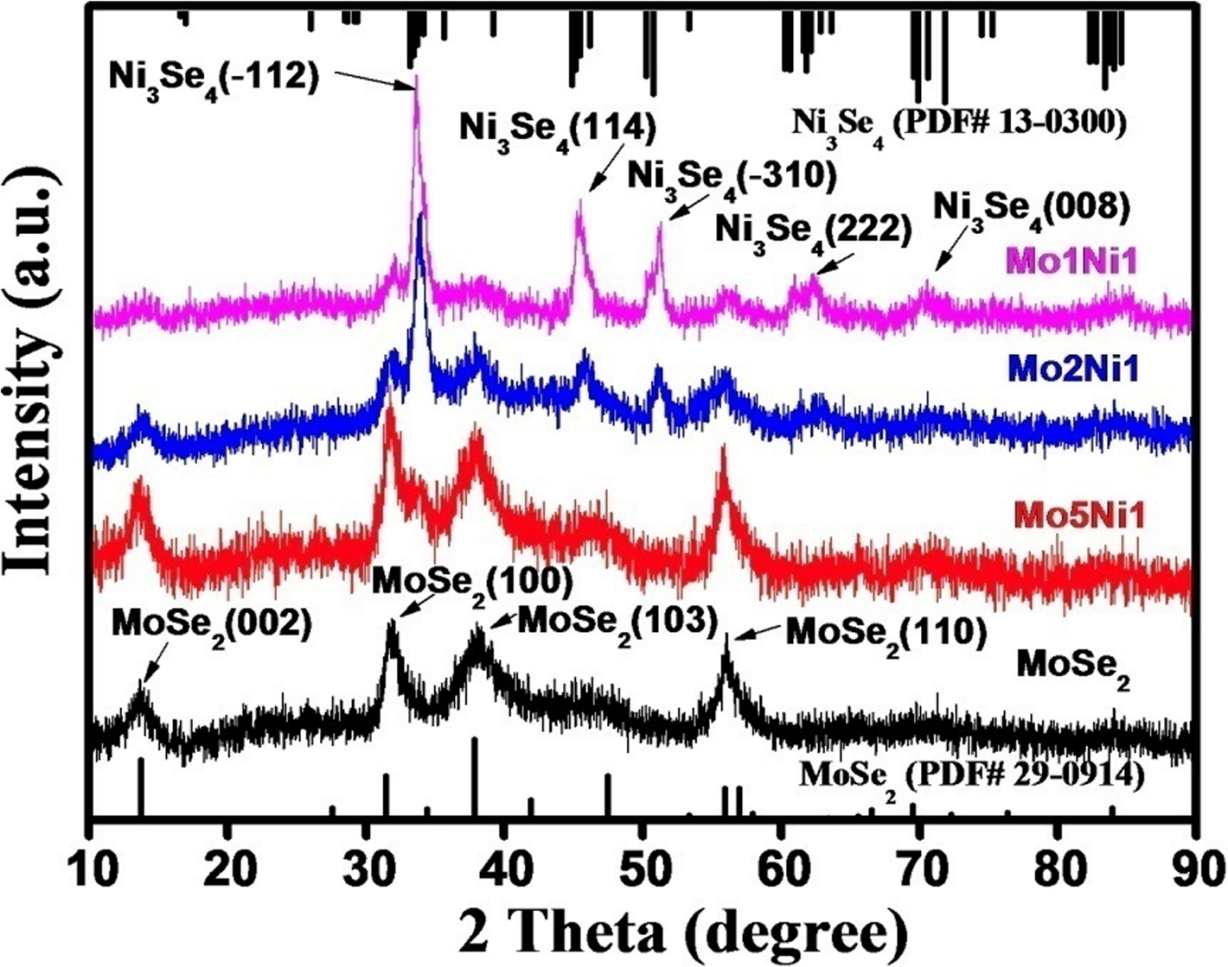
XRD patterns of pure MoSe_2_ and MoSe_2_–Ni_3_Se_4_ hybrid samples with different Mo:Ni ratios. Reference patterns of bulk MoSe_2_ and Ni_3_Se_4_ are also included

Figure 2 compares the XRD patterns of pure MoSe_2_ and MoSe_2_–Ni_3_Se_4_ hybrid samples. The diffraction peaks of pure MoSe_2_ sample are in accordance with hexagonal MoSe_2_ (PDF# 29-0914) while the MoSe_2_–Ni_3_Se_4_ hybrid samples with different Mo:Ni ratios exhibit the combinational peaks of hexagonal MoSe_2_ and monoclinic Ni_3_Se_4_ (PDF# 13-0300). As the content of Ni precursor added increases, the peak intensity of Ni_3_Se_4_ in the XRD patterns also increases, which indicate that the concentration of Ni_3_Se_4_ in the MoSe_2_–Ni_3_Se_4_ hybrid nanoelectrocatalysts increases too. Therefore, the content of Ni_3_Se_4_ in the MoSe_2_–Ni_3_Se_4_ hybrid nanoelectrocatalysts can be tuned by controlling the content of the Ni precursor added. The SAED analyses (Additional file 1: Figure S1) also reveal the co-existence of hexagonal MoSe_2_ and monoclinic Ni_3_Se_4_, which confirm the XRD results. As the content of Ni precursor added increases, the diffraction rings belonging to Ni_3_Se_4_ also become prominent, demonstrating that the relative content of Ni_3_Se_4_ component in MoSe_2_–Ni_3_Se_4_ hybrid nanoelectrocatalysts increases too.

**Fig. 3:**
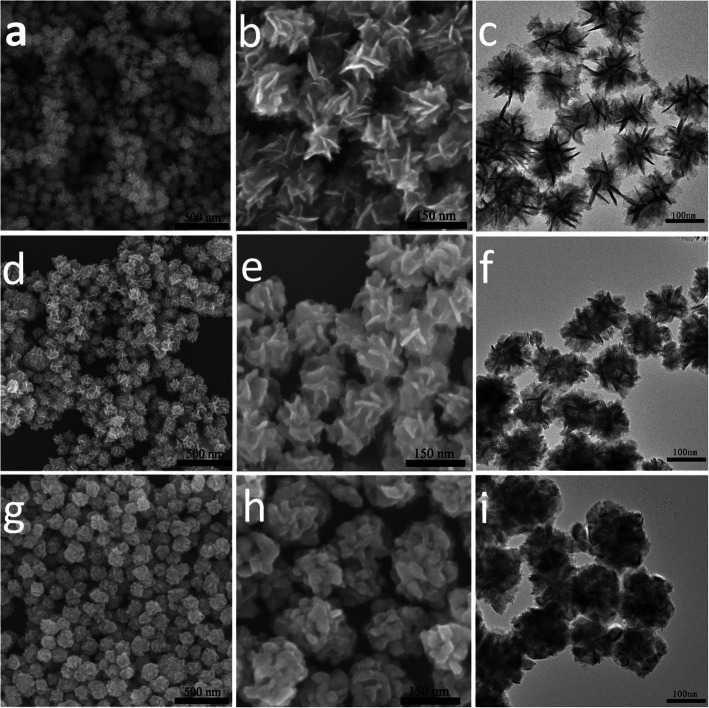
SEM images (**a**, **b**, **d**, **e**, **g**, and **h**) and TEM images (**c**, **f**, and **i**) of Mo5Ni1 (**a**−**c**), Mo2Ni1 (**d**−**f**) and Mo1Ni1 (**g**−**i**) samples

**Fig. 4:**
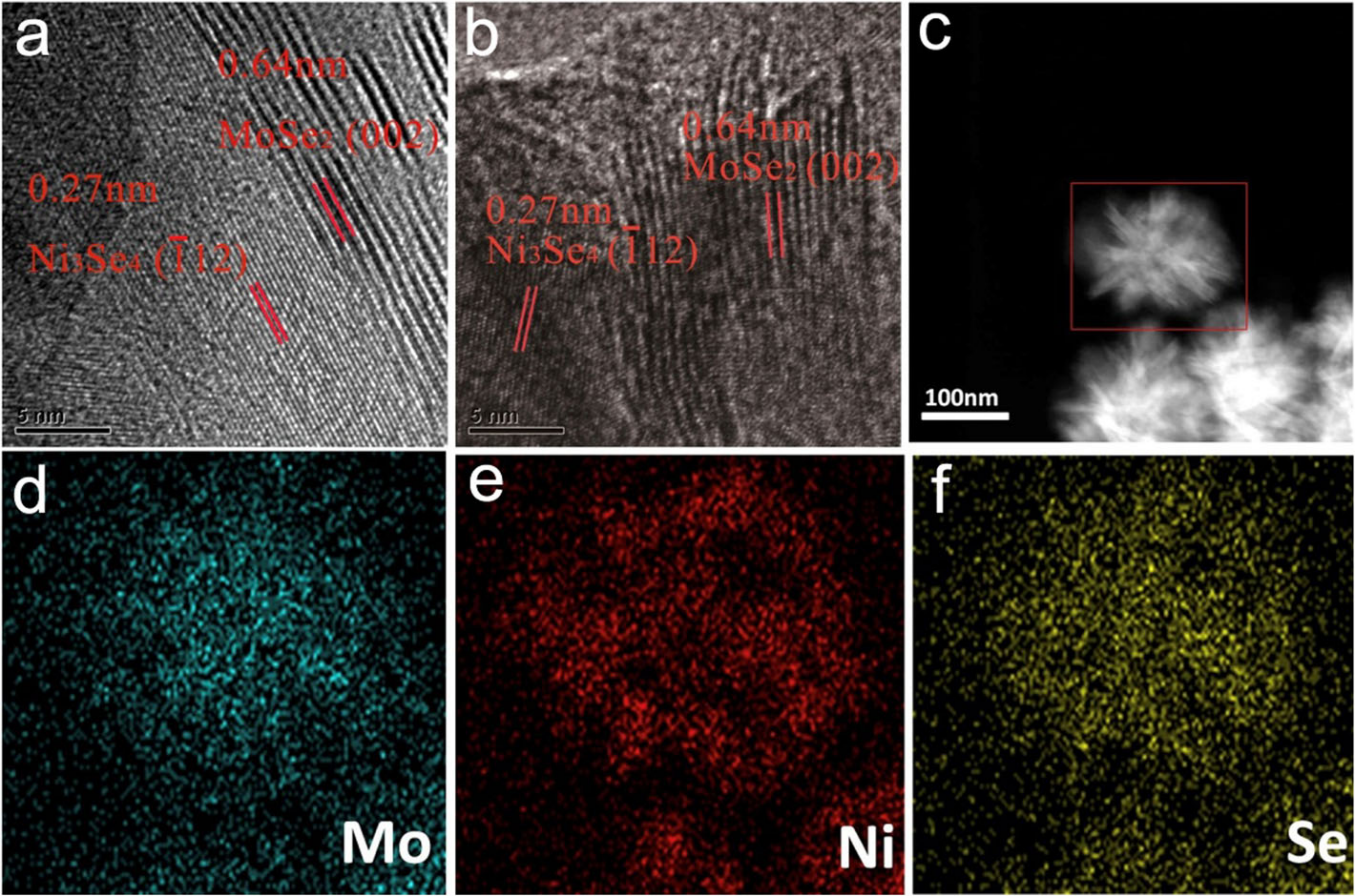
HRTEM images (**a** and **b**), HAADF image (**c**) and elemental maps (**d**−**f**) of Mo2Ni1 sample

The morphology of as-prepared samples was analyzed by SEM and TEM. The pure MoSe_2_ possesses a flower-like morphology that has a size ranging from 100 to 200 nm (Additional file 1: Figure S2). Upon incorporating Ni_3_Se_4_, it can be distinctly seen that the petals of nanoflowers begin to become thicker (Fig. 3), and the flower-like morphology tends to disappear gradually with increasing the Ni_3_Se_4_ content. High-resolution TEM (HRTEM) analyses (Fig. 4a, b) on Mo2Ni1 sample reveal two types of evident lattice fringes: the one having an interplanar spacing of 0.64 nm corresponds to the (002) plane of MoSe_2_ [25], and the one with an interplanar spacing of 0.27 nm agrees well with the (−112) plane of Ni_3_Se_4_. The result confirms the presence of both MoSe_2_ and Ni_3_Se_4_ components in a hybrid nanostructure, and the main surfaces of nanoflower petals are constituted by the {001} facets of MoSe_2_. In addition, the two different lattice fringes are roughly in parallel, indicating that Ni_3_Se_4_ may grow on the {001} facets of MoSe_2_ along the c-axis of MoSe_2_.

Energy-dispersive X-ray spectroscopy (EDS) elemental maps along with the HAADF image (Fig. 4**d**−**f**) confirm the presence of Se, Ni and Mo. However, the spatial distribution of Mo and Ni is slightly different. Mo is basically distributed homogeneously in the nanoflower, whereas Ni tends to concentrate near the petals of the nanoflower, which indicate that Ni_3_Se_4_ should grow on MoSe_2_ petals. The covering of thicker Ni_3_Se_4_ layers on the MoSe_2_ may block the active sites of MoSe_2_ and eventually leads to declined HER performances. Besides to the injected amount of Ni and Se sources, the injection rate also affects the morphology of MoSe_2_–Ni_3_Se_4_ hybrid nanostructure. When a smaller injection rate (1.65 mL/min) of Ni and Se sources was used, the products turned out to have an inhomogeneous morphology (Additional file 1: Fig. S3). This indicates that the formation of MoSe_2_–Ni_3_Se_4_ hybrid nanostructure is also a kinetically controlled process.

**Fig. 5:**
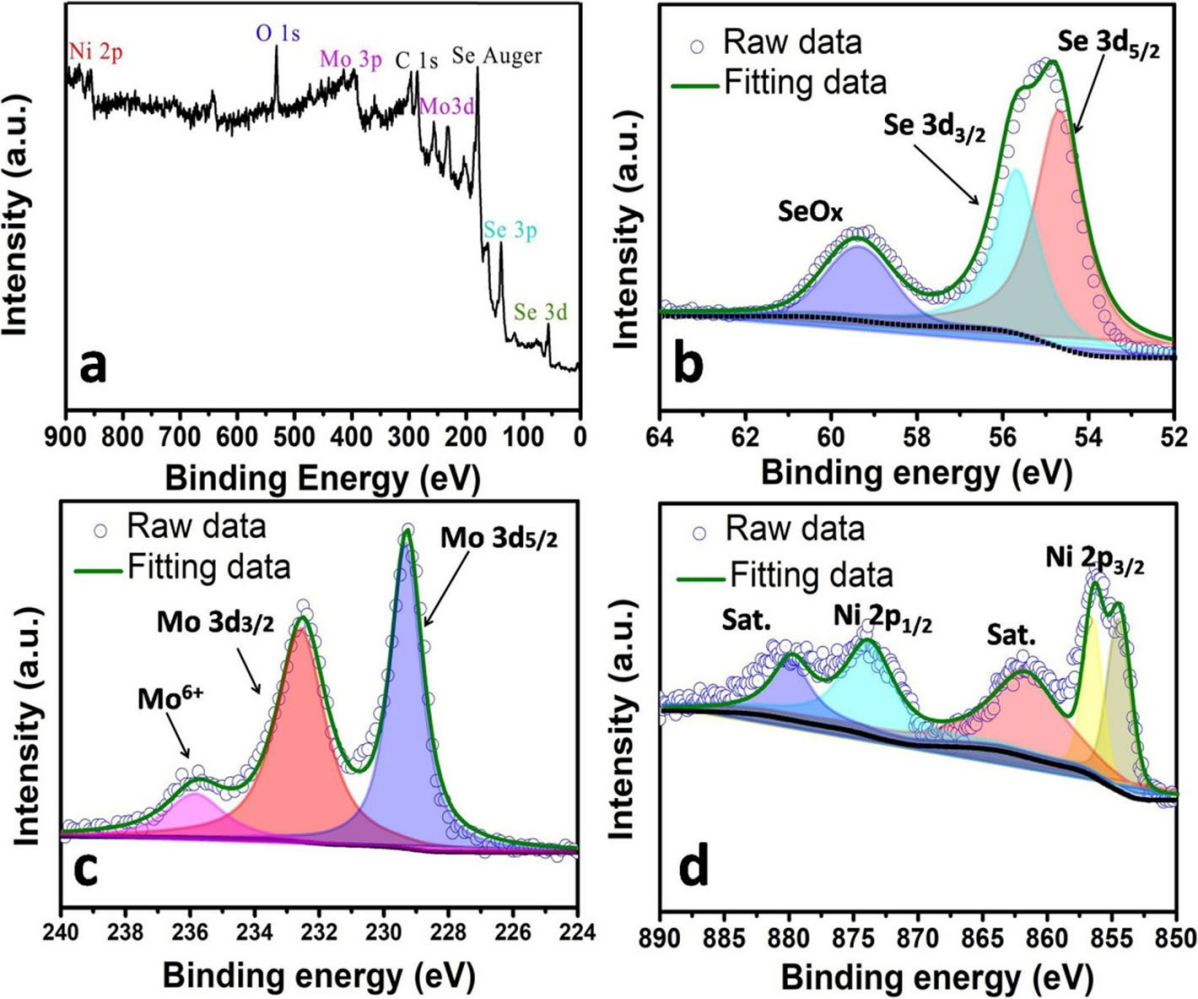
XPS spectra of Mo2Ni1 sample. **a** is the survey spectrum. **b**), **c**, and **d** show the expanded spectra of Se, Mo, and Ni, respectively

XPS analyses (Fig. 5a–d) further verify the presence of Mo, Ni, and Se in the hybrid sample (take Mo2Ni1 as a typical example). For Se 3d regions (Fig. 5b), the two peaks at 54.75 and 55.75 eV are assigned to Se 3d_5/2_ and Se 3d_3/2_, respectively, which indicates that the oxidation state for Se at is − 2 [26]. The obvious peak at 59.37 eV suggests that the Se species at surfaces has been oxidized [20, 26]. In Fig. 5c, two peaks located at 229.37 and 232.50 eV are assigned to Mo 3d_5/2_ and 3d_3/2_, respectively, which indicate the +4 oxidation state of Mo [8, 11, 26]. In Fig. 5d, the Ni 2p peaks are clearly present, and the peaks at 856.62 and 874.12 eV agree well with Ni 2p_3/2_ and Ni 2p_1/2_, respectively. The two satellite peaks at 861.87 and 880.37 eV suggest that Ni is in the oxidation state close to + 2 [27].

The formation mechanism of MoSe_2_–Ni_3_Se_4_ hybrid nanostructure can be understood from the above characterization results. The flower-like MoSe_2_ seeds play an important role in inducing the formation of Ni_3_Se_4_ on the surfaces of MoSe_2_. At the reaction temperature of 300 °C, Ni(acac)_2_ is easily to decompose to react with Se to form Ni_3_Se_4_. The surfaces of MoSe_2_ can act as heterogeneous-nucleation sites to induce the nucleation of Ni_3_Se_4_. Obviously such a heterogeneous nucleation process requires less active energy than homogeneous nucleation. Therefore Ni_3_Se_4_ is observed to grow on the surfaces of MoSe_2_ to form petal-like morphology instead of separated particles which are formed by independently homogeneous nucleation. With further increasing the amounts of Ni and Se sources, Ni_3_Se_4_ tends to grow on the surfaces of Ni_3_Se_4_ petals that have already formed. As a result, MoSe_2_–Ni_3_Se_4_ hybrid nanostructures with increased thickness of Ni_3_Se_4_ petals are observed (see the morphological evolution shown in Fig. 3).

**Fig. 6:**
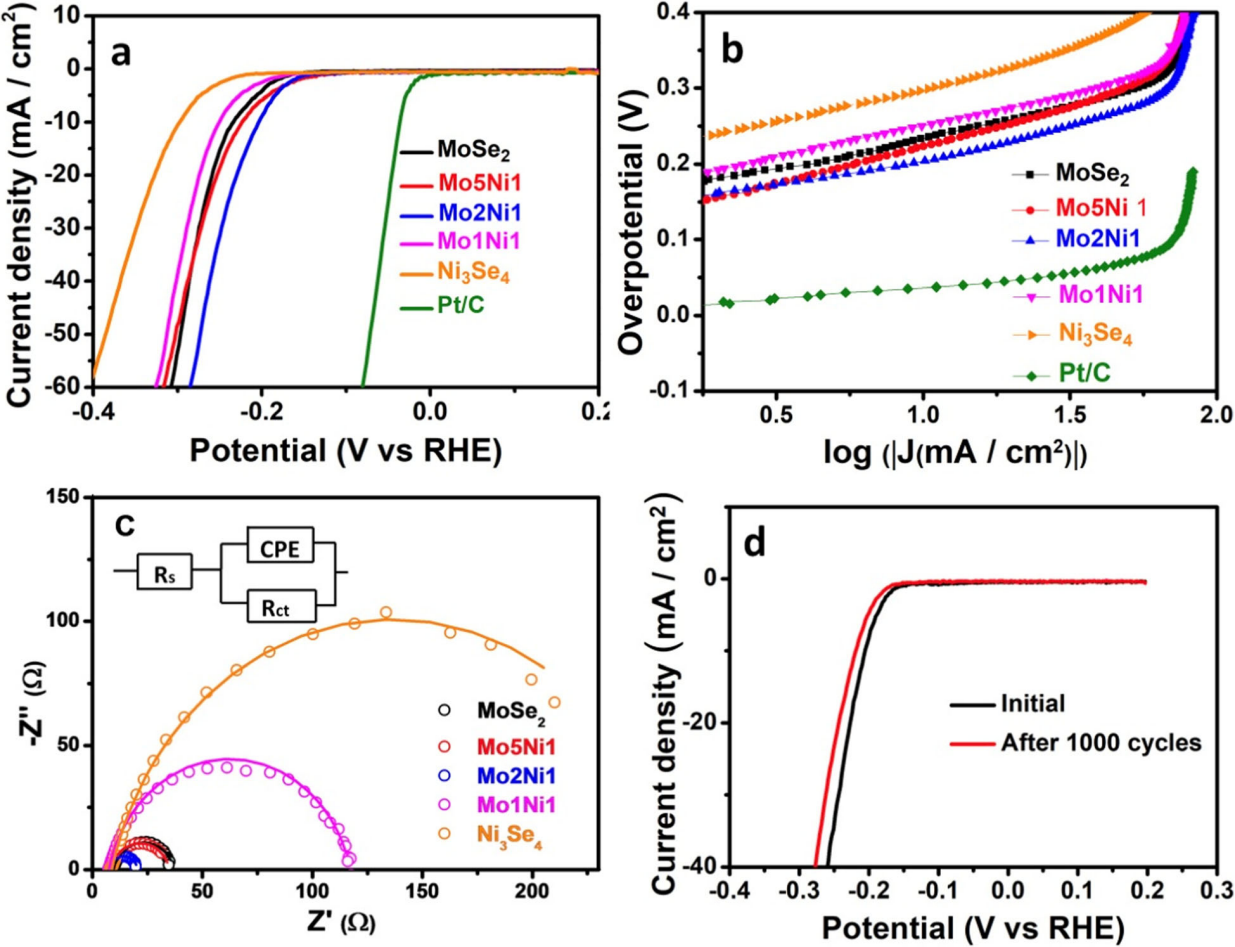
Polarization curves (**a**) and corresponding Tafel plots (**b**) of MoSe_2_, Mo5Ni1, Mo2Ni1, Mo1Ni1, Ni_3_Se_4_, and Pt/C. **c** Nyquist plots at an overpotential of 250 mV. **d** Polarization curves of Mo2Ni1 sample before and after 1000 cycles

The electrocatalytic activity of as prepared catalysts was measured using a three-electrode system in acid solution. As shown in Fig. 6a, all the onset overpotentials (i.e., the potential needed to achieve a current density of 1 mA cm^−2^) [28] of various catalysts are small. The Mo5Ni1 sample requires the lowest onset overpotential of 128 mV for HER, while for other catalysts, the values of onset overpotential are 163, 140, 162, and 216 mV for MoSe_2_, Mo2Ni1, Mo1Ni1, and Ni_3_Se_4_, respectively. When cathode current density reaches -10 mA cm^-2^, the Mo2Ni1 sample requires the smallest overpotential of 203 mV. The needed overpotentials are 234, 220 250, and 299 mV for MoSe_2_, Mo5Ni1, Mo1Ni1, and Ni_3_Se_4_, respectively. To further investigate the obtained samples, the linear portions of the Tafel curves were analyzed using the Tafel equation:

1 $$ \eta =b\;\log\;j+a $$

where *j* is the current density, *η* is the overpotential, and *b* is the Tafel slope. As can be seen in Fig. 6b, the Mo2Ni1 sample has a Tafel slope of 57 mV per decade. This value is substantially smaller than the slopes of Mo5Ni1 (85 mV per decade), Mo1Ni1 (88 mV per decade), Ni_3_Se_4_ (82 mV per decade) and MoSe_2_ (71 mV per decade) samples. Meanwhile, the Pt/C exhibits a Tafel slope of ~ 33 mV per decade, corresponding well to the known values [29]. Theoretically, the lower Tafel slope suggests the faster HER kinetics [30]. The principal reaction mechanism in the HER process can be revealed by the Tafel slope [15, 19]. There are three main steps can participate in the HER process, i.e., Volmer reaction: H^+^ (aq) + e^-^ → H_ads_, Heyrovsky reaction: H_ads_ + H^+^ (aq) + e^-^ → H_2_ (g), and Tafel reaction H_ads_ + H_ads_ →H_2_ (g). At 25 °C, The Tafel slope values of the three reactions are 118 mV per decade, 39 mV per decade, and 29 mV per decade, respectively [19]. Accordingly, the results in our study suggest that the mechanism of Volmer–Heyrovsky [31–33] should be dominant for all prepared samples in the HER.

In order to further investigate the kinetics of electrodes, the Nyquist plots of five samples acquired by EIS are shown in Fig. 6c. The charge transfer resistance (*R*_ct_) which is achieved from the region of low frequency has a close relationship to the kinetics of electrodes. A smaller value of *R*_ct_ is relevant to a higher reaction rate [34]. The value of *R*_ct_ of Mo2Ni1 is 13.0 Ω, which is the lowest value among the five samples. For other samples, the *R*_ct_ values are 27.5, 27.1, 109.1, and 254.6 Ω for MoSe_2_, Mo5Ni1, Mo1Ni1, and Ni_3_Se_4_, respectively. The lowest *R*_ct_ of Mo2Ni1 suggests the fastest charge transfer process among the as prepared samples. The result further proves the excellent HER electrocatalytic efficiency of the Mo2Ni1 sample. The better conductivity might be resulted from the modulation of electronic structure via the synergetic effects between MoSe_2_ and Ni_3_Se_4_. Fig. 6d presents the polarization curves to characterize the stability of Mo2Ni1 sample. After 1000 cycles, the catalytic performance only shows a slight decline. The synergetic effects play an important in controlling the adsorptive-absorptive interactions on the catalytic surfaces and thus determine the rate determining step of the catalytic reaction [35]. Therefore, the utilization of synergetic effects constitutes a major advantage of hybrid nanostructure for the enhancement of HER activity.

**Fig. 7:**
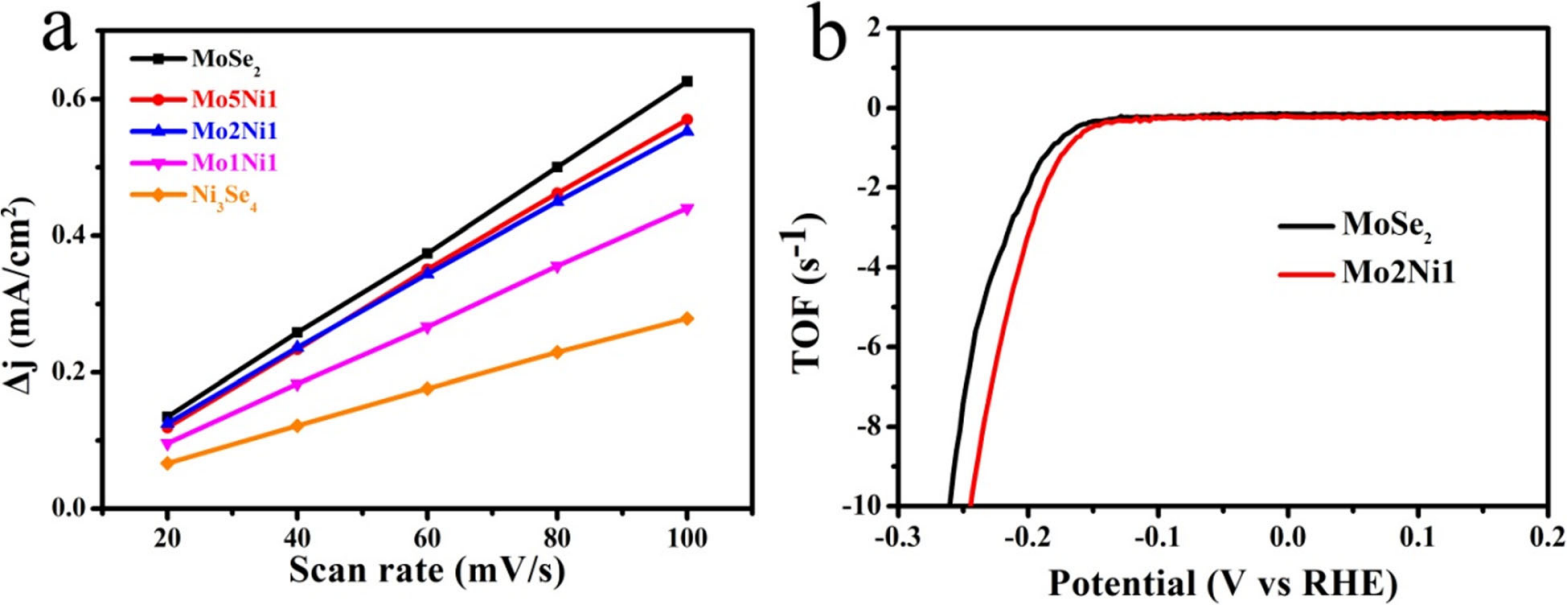
**a** Double-layer capacity current versus scan rate of MoSe_2_, Mo5Ni1, Mo2Ni1, Mo1Ni1, and Ni_3_Se_4_ samples. **b** Calculated TOFs of pure MoSe_2_ and Mo2Ni1 samples

To roughly calculate the electrochemically active surface area (ESCA) of the catalysts, electrochemical double-layer capacitances (*C*_dl_) are measured using cyclic voltammetry (CV) at different scan rates (Additional file 1: Figure S4). The plots of Δ*j* = (*j*_a_−*j*_c_)( *j*_a_ and *j*_c_ are the current density when charging and discharging at a voltage of 0.15 V, respectively) against the scan rate are shown in Fig. 7a, and the *C*_dl_ values are counted to be half of the slopes. Mo2Ni1 exhibits a *C*_dl_ value of 2.67 mF cm^−2^ which is slightly smaller than the value (3.06 mF cm^−2^) of MoSe_2_ and Mo5Ni1 (2.82 mF cm^−2^), suggesting that the addition of Ni_3_Se_4_ cannot further increase the electrochemical active surface area, and the consequence is consistent with the TEM observation. Hence the reason for the improvement of the HER catalytic activity of Mo2Ni1 sample is not likely due to the increase of electrochemically active surface area but the synergistic effect between MoSe_2_ and Ni_3_Se_4_, along with the promoting of conductivity. In addition, we estimated the numbers of active sites and turnover frequencies (TOFs) of various catalysts. The numbers of actives are obtained by the CV curves of different catalysts which are recorded from − 0.4 to 0.6 V in a phosphate buffer saline electrolyte with a scan rate of 50 mV s^−1^(Additional file 1: Figure S5) [30, 36]. The calculated number of active sites for Mo2Ni1 is 1.02 × 10^−6^ mol while that for MoSe_2_ is 0.77 × 10^−6^ mol. In addition, the calculated TOF at – 200 mV for each active site of Mo2Ni1 is 3.4 s^−1^, which is also larger than that (2.1 s^−1^) of MoSe_2_ (Fig. 7b). Theoretically, the HER activity of catalysts can be attributed to three factors: (a) the active site numbers, (b) the active site quality (turnover frequency), and (c) the conductivity among active sites [37]. In this work, although Mo2Ni1 has a slightly smaller value of C_dl_ compared to MoSe_2_, it possesses the lowest charge–transfer impedance, the most active sites and the highest TOF. Therefore, it exhibits the best overall HER activity.

## Conclusions

A seed-induced solution route has been developed for the synthesis of MoSe_2_–Ni_3_Se_4_ hybrid nanoelectrocatalysts. MoSe_2_ seeds with a flower-like morphology that is composed by the assembly of ultrathin nanoflakes have been used to induce the growth of Ni_3_Se_4_ on the flower petals of MoSe_2_. The chemical composition of MoSe_2_–Ni_3_Se_4_ hybrid nanoelectrocatalysts can be modulated by adjusting the content of Ni_3_Se_4_. It has been observed that the combination of Ni_3_Se_4_ with MoSe_2_ to form a hybrid nanostructure can improve the HER performances of MoSe_2_. The MoSe_2_–Ni_3_Se_4_ hybrid nanoelectrocatalyst with a Mo:Ni ratio of 2:1 delivers remarkable HER performances that have a small onset overpotential of 140 mV, an overpotential of 201 mV at 10 mA cm^−2^ and a small Tafel slope of 57 mV dec^−1^ under acidic condition. The improved conductivity and TOF have also been observed.

## Supplementary information

**Additional file 1: Fig. S1** SAED patterns of Mo5Ni1 (a), Mo2Ni1 (b) and Mo1Ni1 (c) samples. The indexes in white correspond to Ni_3_Se_4_ while those of red correspond to MoSe_2_. **Fig. S2** SEM image (a) and TEM image (b) of pure MoSe_2_. **Fig. S3** TEM images of Mo2Ni1 sample obtained using different injection rates. (a) 3.3 mL/min. (b) 1.65 mL/min. (c) XRD patterns (The bottom pattern corresponds to an injection rate of 1.65 mL/min while the up one to 3.3 mL/min). **Fig. S4** Cyclic voltammetry curves of pure (a) MoSe_2_, (b) Mo5Ni1, (c) Mo2Ni1, (d) Mo1Ni1 and (e) pure Ni_3_Se_4_ in the region of 0.1 ~ 0.2 V vs RHE. **Fig. S5** Cyclic voltammograms (-0.1~0.6 V vs RHE) recorded in pH = 7 phosphate buffer.

## Data Availability

Not applicable.

## Abbreviations

HER: Hydrogen evolution reaction
XRD: X-ray diffraction
TMDs: Transition metal dichalcogenides
SEM: Scanning electron microscopy
TEM: Transmission electron microscopy
SAED: Selected area electron diffraction
HAADF: High-angle annular dark-field
XPS: X-ray photoelectron spectroscopy
EIS: Electrochemical impedance spectroscopy
TOFs: Turnover frequencies
*R*_ct_: Charge transfer resistance
*C*_dl_: Electrochemical double-layer capacitances
