# Supplementary material for: MoSe2-Ni3Se4 Hybrid Nanoelectrocatalysts and Their Enhanced Electrocatalytic Activity for Hydrogen Evolution Reaction
Source: Nanoscale Res Lett. 2020 Jun 16;15:132. doi: 10.1186/s11671-020-03368-z (PMC7297896; doi:10.1186/s11671-020-03368-z)
Supplement: Supplementary file 1 — Additional file 1: Fig. S1 SAED patterns of Mo5Ni1 (a), Mo2Ni1 (b) and Mo1Ni1 (c) samples. The indexes in white correspond to Ni3Se4 while those of red correspond to MoSe2. Fig. S2 SEM image (a) and TEM image (b) of pure MoSe2. Fig. S3 TEM images of Mo2Ni1 sample obtained using different injection rates. (a) 3.3 mL/min. (b) 1.65 mL/min. (c) XRD patterns (The bottom pattern corresponds to an injection rate of 1.65 mL/min while the up one to 3.3 mL/min). Fig. S4 Cyclic voltammetry curves of pure (a) MoSe2, (b) Mo5Ni1, (c) Mo2Ni1, (d) Mo1Ni1 and (e) pure Ni3Se4 in the region of 0.1 ~ 0.2 V vs RHE. Fig. S5 Cyclic voltammograms (-0.1~0.6 V vs RHE) recorded in pH = 7 phosphate buffer. [file 11671_2020_3368_MOESM1_ESM.docx]

**Supporting Information**

**MoSe_2_-Ni_3_Se_4_ hybrid nanoelectrocatalysts and their enhanced electrocatalytic activity for hydrogen evolution reaction**

Pengyuan Wu, Gangyong Sun, Yuanzhi Chen*, Wanjie Xu, Hongfei Zheng, Jin Xu*, Laisen Wang and Dong-Liang Peng

*Department of Materials Science and Engineering, Collaborative Innovation Center of Chemistry for Energy Materials, College of Materials, Xiamen University, Xiamen 361005, China*


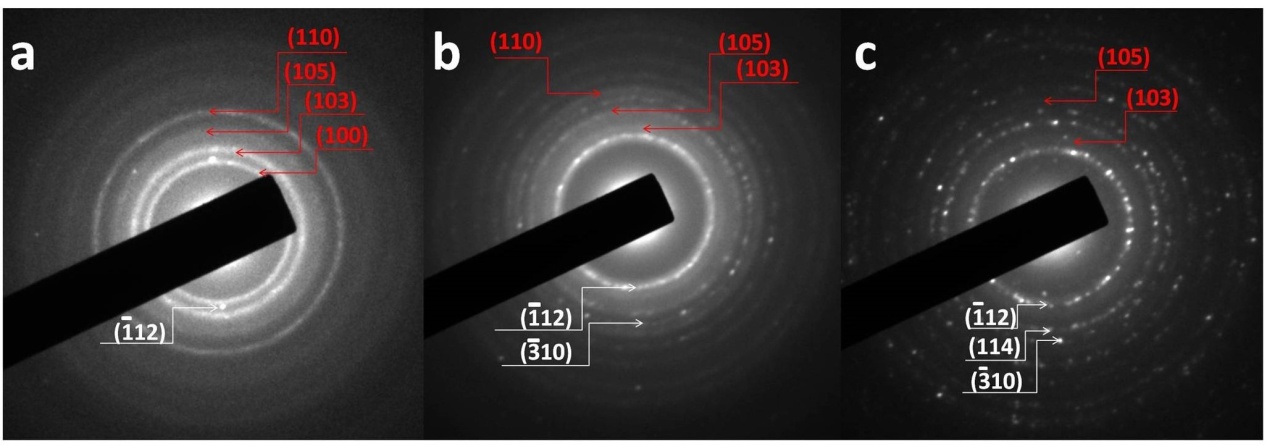


**Fig. S1** SAED patterns of Mo5Ni1 (a), Mo2Ni1 (b) and Mo1Ni1 (c) samples. The indexes in white correspond to Ni_3_Se_4_ while those of red correspond to MoSe_2_.


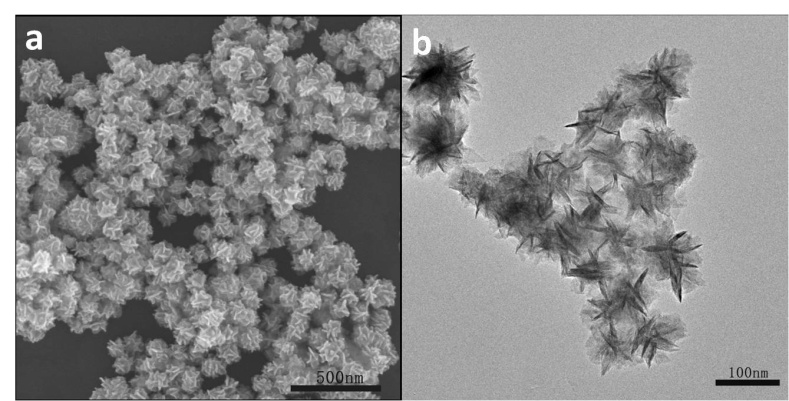


**Fig. S2** SEM image (a) and TEM image (b) of pure MoSe_2_.


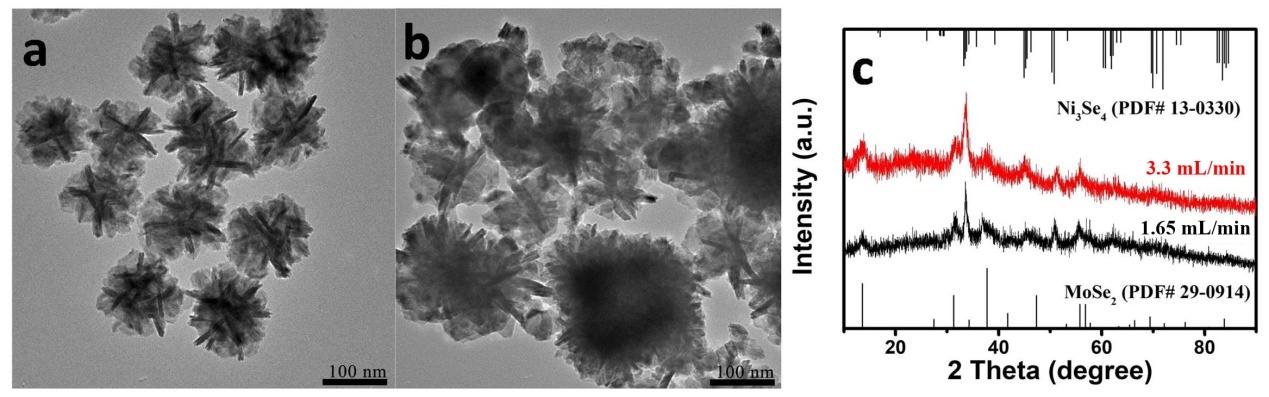


**Fig. S3** TEM images of Mo2Ni1 sample obtained using different injection rates. (a) 3.3 mL/min. (b) 1.65 mL/min. (c) XRD patterns (The bottom pattern corresponds to an injection rate of 1.65 mL/min while the up one to 3.3 mL/min).


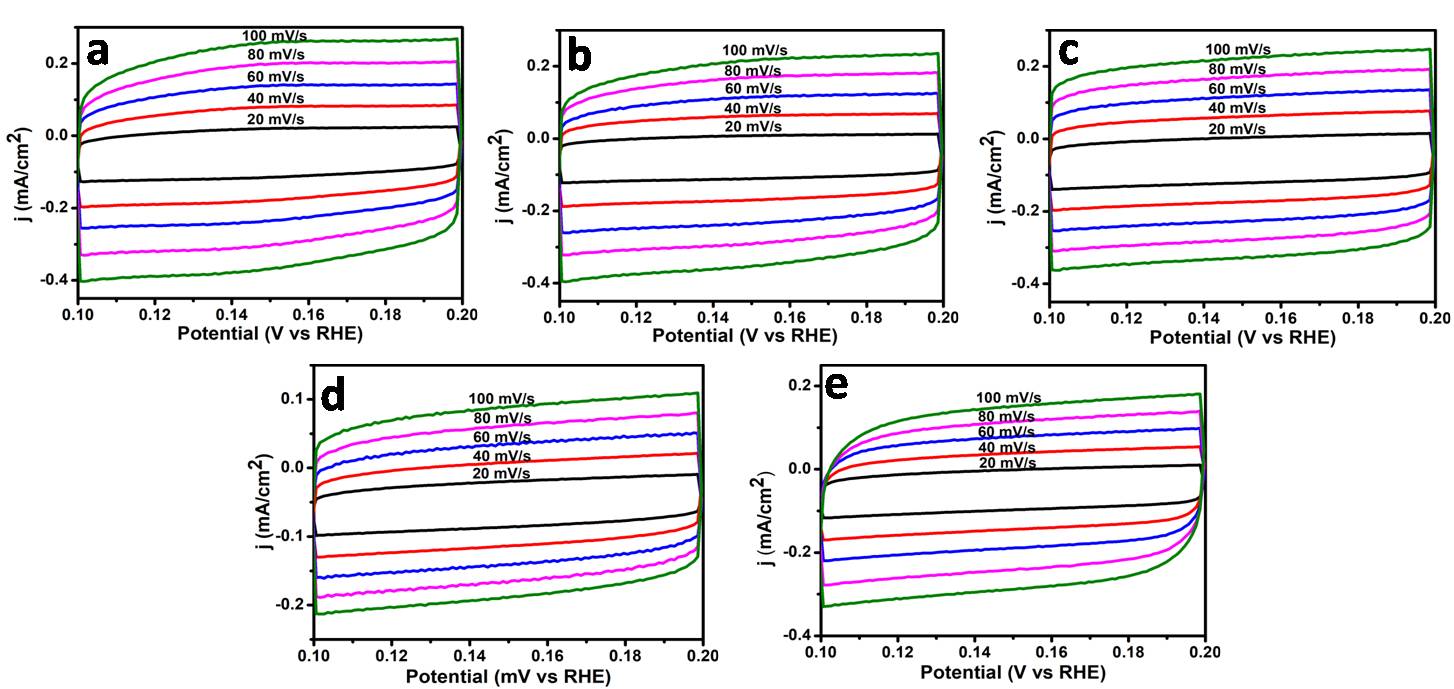


**Fig. S4** Cyclic voltammetry curves of pure (a) MoSe_2_, (b) Mo5Ni1, (c) Mo2Ni1, (d) Mo1Ni1 and (e) pure Ni_3_Se_4_ in the region of 0.1 ~ 0.2 V vs RHE.


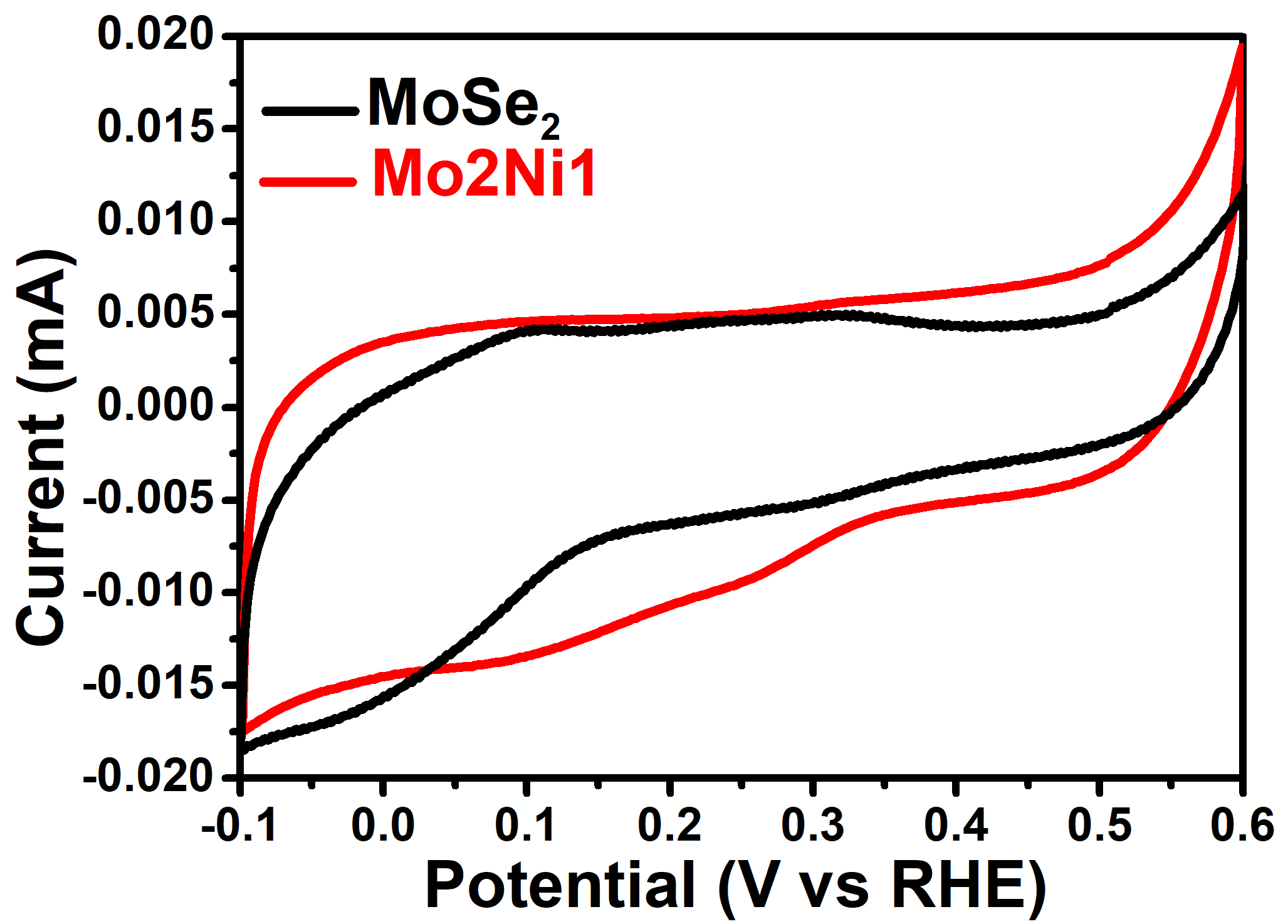


**Fig. S5** Cyclic voltammograms (-0.1~0.6 V vs RHE) recorded in pH = 7 phosphate buffer.
